# TiHoVideos: veterinary students’ utilization of instructional videos on clinical skills

**DOI:** 10.1186/s12917-019-2079-2

**Published:** 2019-09-11

**Authors:** Lina R. Müller, Andrea Tipold, Jan P. Ehlers, Elisabeth Schaper

**Affiliations:** 1 E-Learning Department, University of Veterinary Medicine Hannover, Bünteweg 2, 30559 Hannover, Germany; 2 Small Animal Clinic, Neurology, University of Veterinary Medicine Hannover, Bünteweg 9, 30559 Hannover, Germany; 3 University Witten/Herdecke, Alfred-Herrhausen-Strasse 50, 58448 Witten, Germany

**Keywords:** Veterinary education, E-learning, Distance learning, Educational video, Clinical skills, Students

## Abstract

**Background:**

The YouTube channel “TiHoVideos” was created by the University of Veterinary Medicine Hannover, Foundation (TiHo) to enable easy, public access to the university’s instructional videos as an additional support for learning clinical skills. Video production is expensive and time-consuming. To be able to optimize video production and aligning content to student needs we wanted to know if and how our students use these videos.

**Results:**

Results show that the participating students primarily prepared for learning stations in the Clinical Skills Lab (CSL) by watching TiHoVideos at home on tablets or laptops and then concentrated at the CSL on learning the practical skills hands on. The videos available on TiHoVideos are rated as being a “very helpful” educational tool when preparing for CSL learning stations.

**Conclusions:**

Instructional videos represent an unquestionably suitable medium to aid veterinary students learn practical skills and a contribution to animal welfare by reducing the use of live animals in undergraduate veterinary education. The university’s production of educational video material proves to be worth the effort because the videos are being used, appreciated and well-rated by TiHo students for their learning experience.

**Supplementary information:**

**Supplementary information** accompanies this paper at 10.1186/s12917-019-2079-2.

## Background

As part of a project on quality in teaching funded by the German Federal Ministry of Education and Research, the University of Veterinary Medicine Hannover, Foundation (TiHo) established a Center for Clinical Skills – Clinical Skills Lab (CSL) in 2013 to improve veterinary education in the area of practical skills and the treatment of companion and farm animals. The CSL was set up with the intention of supporting the acquisition of skills with a variety of educational materials [1]. Learning stations focusing on different practical skills were created for this purpose. During the first funding phase of the project, 24 different learning stations were set up and equipped with accompanying educational materials, detailed instructions for the stations, posters and example cases. Accompanying instructional videos were created for the CSL learning stations to help students prepare in advance for practical exercises, check their progress and practice their skills through repetition [1]. To make these instructional videos easy to access, the university launched its own YouTube channel called TiHoVideos (https://www.youtube.com/user/TiHoVideos) [2]. Many videos have been translated through cooperation among universities and are available in English [3], Russian, Chinese, Polish, Estonian and Spanish.

YouTube is also used in medicine as a platform for instructional videos on clinical skills because the publication of open-access resources on this portal can reach a broad audience and enable the compilation of extensive user data [4]. Hibbert [5] determined that the use of high-quality educational videos can significantly improve clinical skills, video material lasts for a long time and can be made available to a large audience of learners at low cost.

In 2013 over 100 h of video material was uploaded onto YouTube [6] each minute, since then no exact numbers have been given by YouTube but it can be assumed that uploaded data increased enormously. Ninety-nine percent of the adolescents and young adults in Germany have currently access to the internet, with YouTube being the most popular content in 2017 [7].

According to different studies analyzing the content of instructional videos on medical topics on YouTube teachers and universities are advised to produce high-quality videos and refer to suitable materials [8–11]. Furthermore, media competence is vital in order to filter for relevant online content [9]. According to Roshier et al., veterinary students prefer high quality instructional videos in terms of sound, availability and content [12]. While Duncan et al. and Rössler et al. criticized the quality and content of educational videos on clinical skills in reference to one or more practical skill [10, 13], our current study proves a positive rating of the university’s videos on YouTube.

Although it was standard for adolescents and young adults to have their own mobile phone in 2012 when the project started, only 47% actually had a smartphone [14]. In 2012, 71% regularly used video portals such as YouTube several times each week; in 2017 the number using YouTube to this extent had grown to 88% [7]. Duncan et al. [13] evaluated the quality and content of instructional video materials on YouTube in terms of clinical skills in December 2011 and found that the videos were often poor and should be viewed critically. The percentage of educationally suitable videos on YouTube at that point in time was low; the suitability of instructional videos can be more easily assessed using criteria defined in advance [15]. Presently, YouTube has more than 1,000,000,000 users, a number that corresponds to almost a third of all Internet users worldwide, and receives billions of views. Most use is by 18-to 34-year-olds [16]. In 2014 YouTube created the channel YouTube EDU which can also be found at #Bildung (#education) [17], where popular educational films have been compiled, including from YouTube partner channels. The channel is administered and added to automatically. Nonetheless, YouTube is viewed critically as an open-learning resource due to the great variability of the content [18]. Therefore, the TiHo created its own channel to guide the students to watch video material produced by their own university’s teachers.

The use of the online platform YouTube was chosen for its popularity among young adults [7]. The user data for TiHo’s YouTube channel show that the number of views for TiHoVideos is steadily climbing and that the videos are used worldwide [19]. However, the utilization of the YouTube channel by TiHo students is not reflected in the analysis of the number of views from Germany alone. For this reason one of this study’s aims was to examine actual use by TiHo students who form the main target group.

We hypothesized that high resource costs in producing educational video material are justified because usage and acceptance amongst veterinary students is high. This is an important information not only for the TiHo but for the wider educational community regarding the need for high quality learning resources.

Another aim was to determine future needs in order to optimally align the production of videos with student needs. Therefore, this study investigates whether the instructional videos on the YouTube channel are utilized by TiHo students, whether this is connected with the use of the CSL, and whether the videos are felt to be a helpful learning tool.

## Results

### Survey

**Fig. 1:**
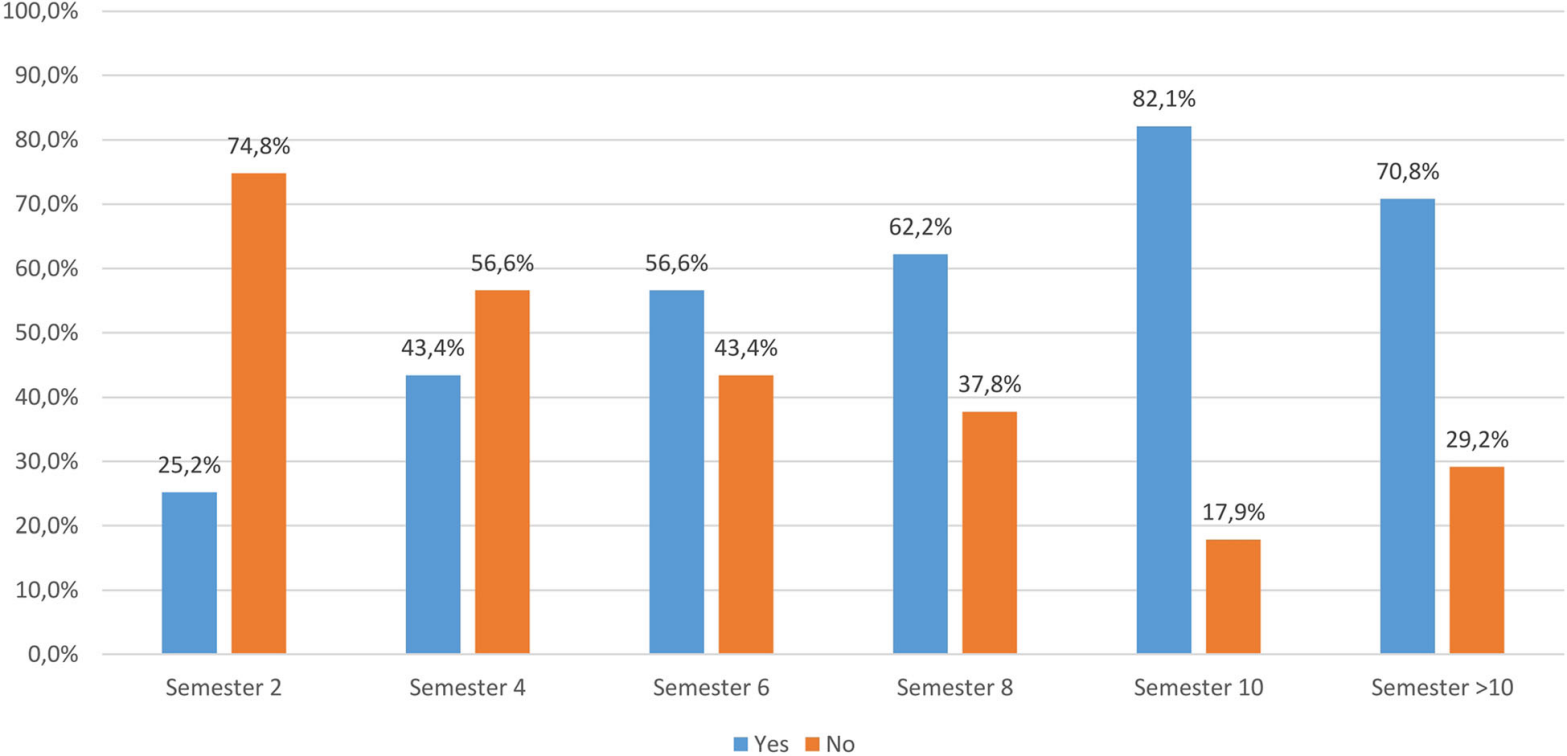
Survey of students at the University of Veterinary Medicine Hannover, Foundation. Question: Have you already used the CSL? (responses: *n* = 833)

The German curriculum encompasses 5 1/2 years and 11 semesters, the 9th and 10th semester are for clinical rotations and practical training whereas the 11th semester is for final examinations. Enrollment is possible once per year in the winter semester starting October, therefore the gathered data is focused on even numbered semesters. The paper-based survey was filled out by 805 students denoting a response rate of 61.8% among the 1302 enrolled students. Thirty students additionally participated in the online survey. A total of 835 students participated to yield an overall response rate of 63.5%, see Table 1. When asked if they had already used the CSL, more students in the second and fourth semester responded negatively than positively. In the higher semesters more students had used the CSL than had not. Overall, 387 of the surveyed students had already used the CSL (46.5%) (Fig. 1).

**Table 1:** Total response rate of survey, total responses *n* = 835

Semester	Responses (n)	Response rate (%)
2	242	91.7
4	213	74
6	190	78.5
8	135	53.8
10	28	11.8
> 10	24	
no answer	3

**Fig. 2:**
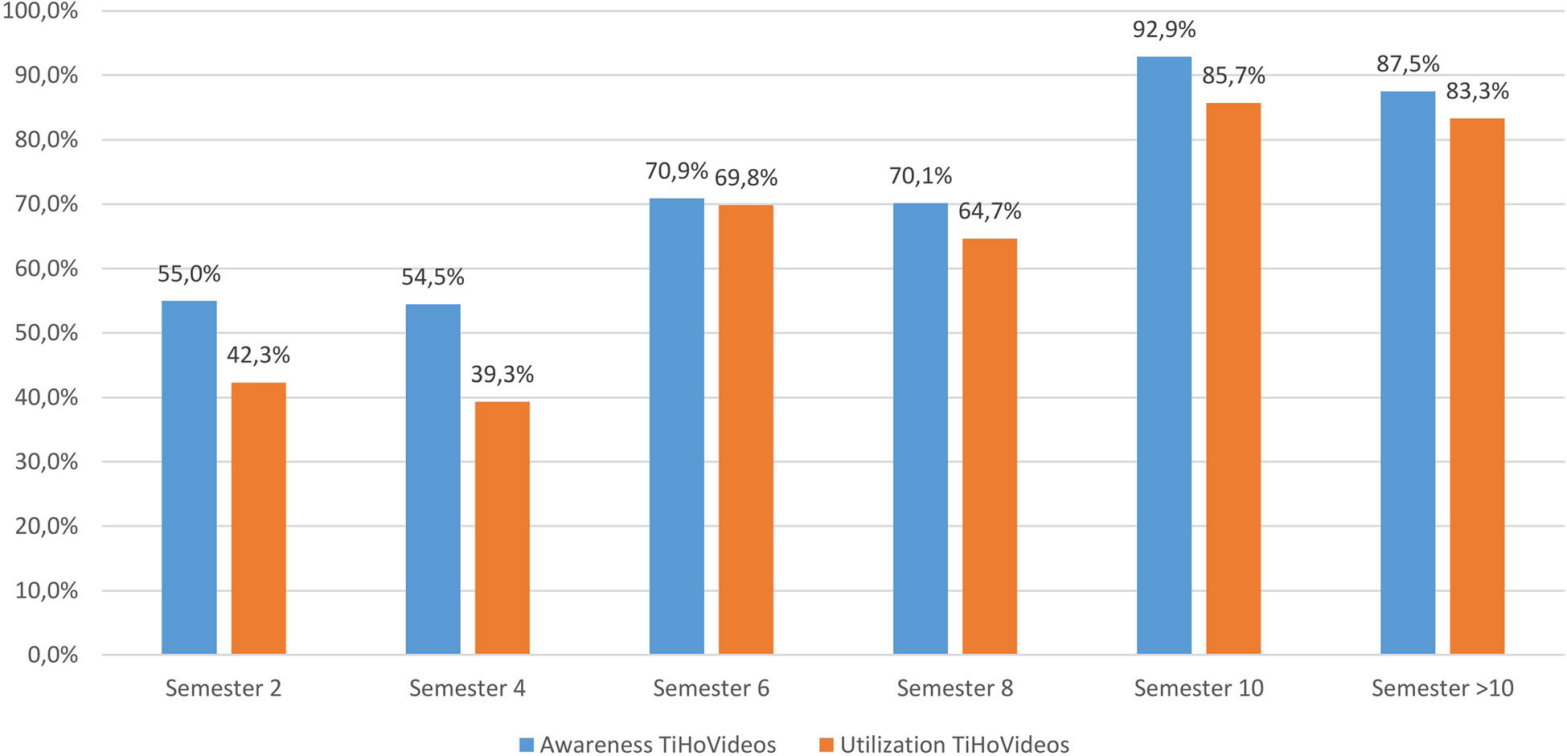
Survey of students at the University of Veterinary Medicine Hannover, Foundation. Questions: Are you aware of the TiHo YouTube channel called TiHoVideos? (responses: *n* = 833) | Have you already viewed videos on TiHoVideos? (responses: *n* = 829)

Responses to the question about familiarity with TiHoVideos showed that students from all semesters had knowledge of the YouTube channel. The degree of familiarity with the channel was higher for all students than the actual utilization. More students in the more advanced semesters used the channel than did not (Fig. 2). 54.2% of the students said that they had watched the videos.

When asked which videos the students had seen on the TiHoVideos channel, students most frequently mentioned *Zwangsmaßnahmen Hund I (Handling, canine I)*, followed by *Zwangsmaßnahmen Katze (Handling feline); Zwangsmaßnahmen Hund II (Handling canine II); Knotentechnik (Knot tying techniques); Intravenöse Injektion Hund (Intravenous injection in dogs); Neurologische Untersuchung Hund (Neurological examination of the dog); U-Heft (U-suture); Subkutane Injektion (Subcutaneous injection); Pfotenverband (Paw bandage), and Donati-Heft (Suture technique Donati).* The possible responses to these questions were compiled in blocks for further analysis (Table 2). Block 6: Surgery/Suturing techniques was mentioned most often by all student groups, followed by block 1 (Animal handling and restraint techniques) and 3 (Venipuncture/Injection). Block 8 (Tutorials) was mentioned least.

**Table 2:** Videos watched by TiHo students – multiple answers possible

Block	Title	Possible responses (original title)	Mentions
1	Animal handling and restraint techniques	Handling, canine I (Zwangsmaßnahmen Hund I)	189
		Handling, canine II (Zwangsmaßnahmen Hund II)	140
		Handling, feline (Zwangsmaßnahmen Katze)	159
2	Bandaging techniques	Head bandage (Kopfverband)	67
		Paw bandage (Pfotenverband)	110
		Bandage thorax/abdomen (Verband Thorax/Abdomen)	69
3	Venipuncture/Injection	Subcutaneous injection (Subcutane Injektion)	112
		Intramuscular injection (Intramuskuläre Injektion)	95
		Intravenous injection in dogs (Intravenöse Injektion Hund)	128
		Intravenous injection in cats (Intravenöse Injektion Katze)	62
		Intravenous injection in model (Intravenöse Injektion Simulator)	52
		Intravenous injection cattle/simulator (Intravenöse Injektion Rind/Simulator)	36
		Insertion of venous catheters and perfusion, cattle (Legen von Venenverweilkatheter und Infusion Rind)	56
		Suboccipital puncture to gain cerebrospinal fluid (Subokzipitale Liquorpunktion)	10
		Implantation and reading transponder chips (Implantation und Auslesen Transponderchip)	32
4	First aid	Intubation in a model (Intubation am Modell)	51
		Resuscitation (Reanimation)	44
5	Hygiene/Sterile procedures	Hand washing (Händewaschung)	64
		Hand disinfection (Händedesinfektion)	50
		Putting on sterile gloves (Anziehen steriler Handschuhe)	58
		Putting on sterile gloves in surgical scrubs (Anziehen steriler Handschuhe im OP-Kittel)	46
6	Surgery/Suturing techniques	Sterile suture removal (Sterile Fadenentnahme)	56
		Removal of needle-thread combination (Entnahme einer Nadel-Faden-Kombination	42
		Knot tying techniques (Knotentechnik)	129
		Instrument knot (Instrumentenknoten)	71
		One-handed surgical knot (Einhandknoten (chirurgisch))	96
		One-handed knot (square knot) (Einhandknoten (Schifferknoten))	83
		Suture technique (Donati) (Donati-Heft)	105
		Simple interrupted stitch (Knopfheft)	103
		Suture technique (Sultan) (Sultansches Diagonalheft)	101
		U-suture (U-Heft)	117
		Suture technique (Bühner) (Bühner-Naht)	49
		Suture technique (Cushing) (Cushing-Naht)	40
		Intracutaneous suture (Intrakutannaht)	59
		Suture technique (Lembert) (Lembertnaht)	63
		Horizontal mattress suture (Kürschnernaht horizontal)	72
		Vertical mattress suture (Kürschnernaht vertikal)	54
		Suture technique (Schmieden) (Schmiedennaht)	53
		Subcutaneous sutures (Subkutannaht)	54
7	Surgical instruments	Preparing basic surgical instruments (Bereitlegen OP Grundbesteck)	37
		Holding surgical instruments (Halten chirurgischer Instrumente)	26
		Scalpel handling (Handhabung Skalpell)	25
		Surgical instruments, abdominal surgery (OP Besteck Bauch OP)	20
8	Tutorials	How to: Start with CASUS (How to: start with CASUS)	22
		CASUS registration (Anmeldung CASUS)	34
9	Information on exams	Electronic assessment at TiHo (Ablauf von elektronischen Prüfungen an der TiHo)	47
		Avoiding cues in multiple-choice questions (Vermeiden von Lösungshinweisen in MC-Fragen)	23
10	Neurological examinations	Neurological examination of the dog (Neurologische Untersuchung Hund)	122
		Neurological examination of the cat (Neurologische Untersuchung Katze)	63
11	Mastitis diagnostics	Udder examination and taking milk samples (Euteruntersuchung und Milchprobennahme)	65
		California Mastitis Test (California Mastitis Test)	87
		Quarter milk samples (Viertelgemelksprobe)	37
12	Other	Study: paraplegic dogs (Patientenstudie querschnittsgelähmte Hunde)	14
		The first days of practicing veterinary medicine (Die ersten Versuche eines Tiermediziners)	11
		Feed selection, goats (Futterselektion Ziege)	24
		Thyroid medicine (Thyreoid Medicine)	1
		Seasonal pastures (Wiese während der Jahreszeiten)	11
		Schmallenberg virus (Schmallenbergvirus)	38

Legend: Survey asking students at the University of Veterinary Medicine Hannover, Foundation (*n* = 835). Responses to the question: Which videos did you watch? – multiple answers possible

**Fig. 3:**
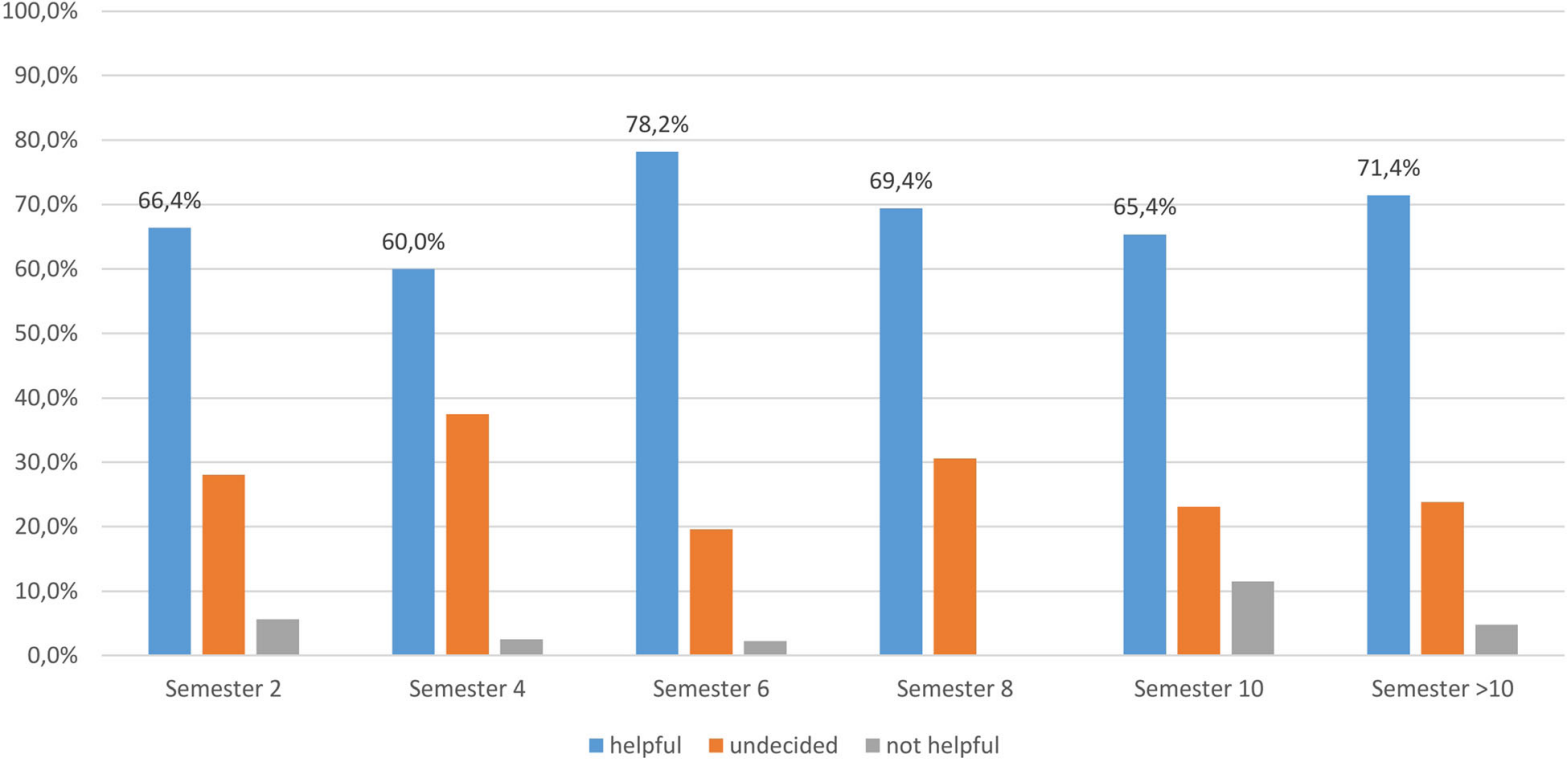
Survey of students at the University of Veterinary Medicine Hannover, Foundation. Question: How helpful are the videos on TiHoVideos for you when learning? helpful = extremely helpful + very helpful, undecided = rather helpful + rather not helpful, not helpful = not helpful + not at all helpful (responses: *n* = 454)

Not all of the students surveyed indicated that they had watched the videos; however, on a six-point Likert scale, 54.4% (*n* = 454) of all students rated the suitability of the videos as learning tools to be on average very helpful (Ø 2.2) (Fig. 3).

In response to the question about frequency of video use, all semester levels reported viewing the videos mainly sporadically: 52.7% in the second, 51.1% in the fourth, 74.1% in the sixth, 73.1% in the eighth, 77.8% in the tenth and 82.6% above the tenth semester. While many students in the second (41.3%) and fourth semester (40.3%) never watched the videos, fewer students answered with “never” in the sixth (17.7%), eighth (23.1%), tenth (14.8%) and above tenth semester (17.4%). Only a few students chose “daily”, “weekly” or “monthly” (*n* = 641).

**Fig. 4:**
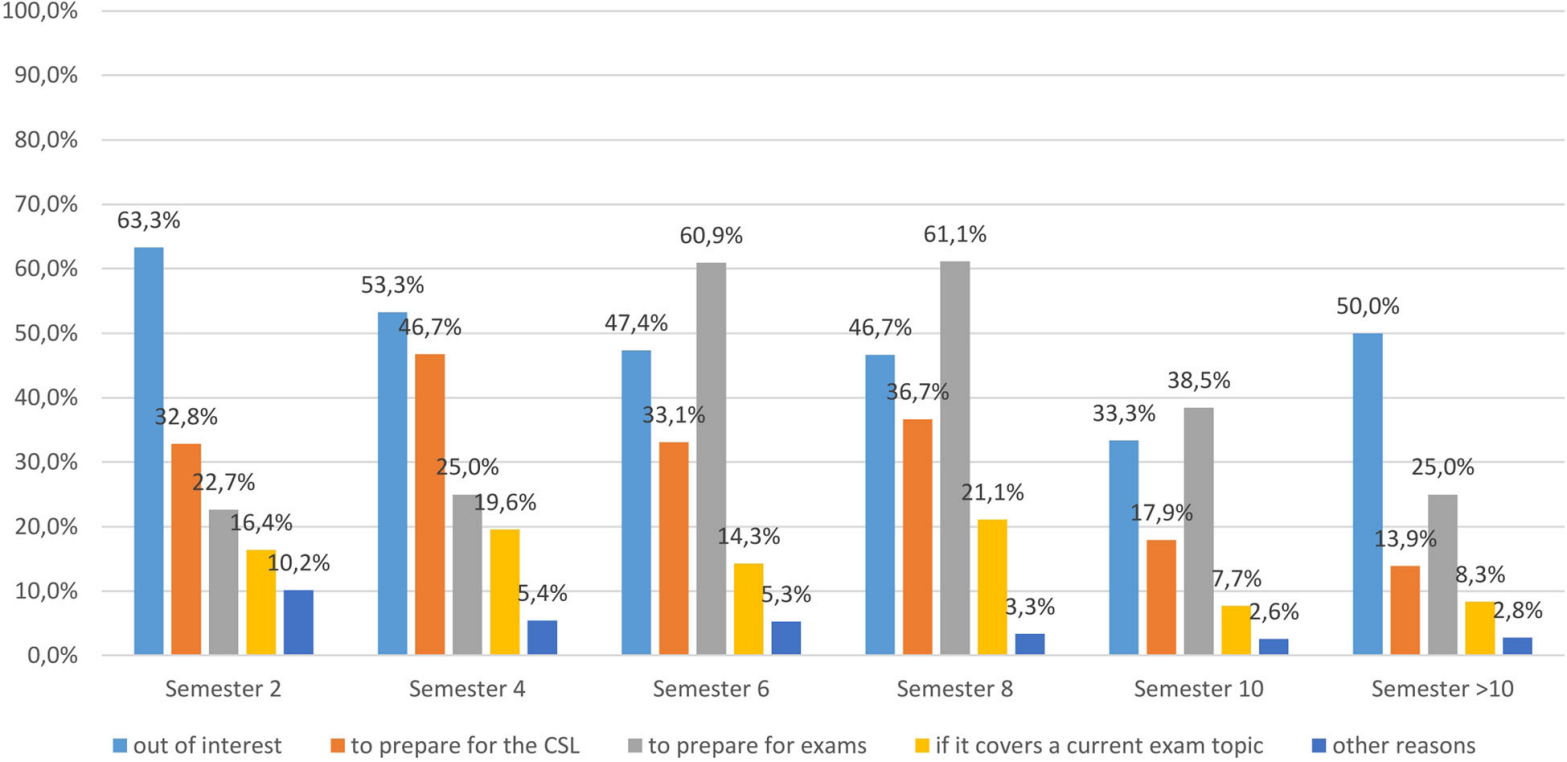
Survey of students at the University of Veterinary Medicine Hannover, Foundation (*n* = 835). Question: Why do you watch videos on TiHoVideos?

A total of 62.5% of students (*n* = 522) responded to the question, why do you watch videos on TiHoVideos? During the second, fourth and after the tenth semester the reason for viewing TiHoVideos is mainly “out of interest” and starting with the sixth semester onward “to prepare for exams”, followed by “out of interest”, “to prepare for the CSL”, “if it covers a current exam topic” and “other reasons” (Fig. 4). The additional reasons that were given include professional relevance (*n* = 4), fostering understanding (*n* = 3), undergoing training to become a veterinary technician (*n* = 2), preparation for exercises (*n* = 2) and lack of alternatives, boredom and preparation for practical exercises (*n* = 1).

A total of 516 (61.8%) students responded to the question about where they watched videos on TiHoVideos. The majority of these students in all semester levels (96.7%) reported watching “at home on a PC”, followed by “mobile” (8.3%), at a TiHo-PC (2.5%) and at the CSL (1.2%).

**Fig. 5:**
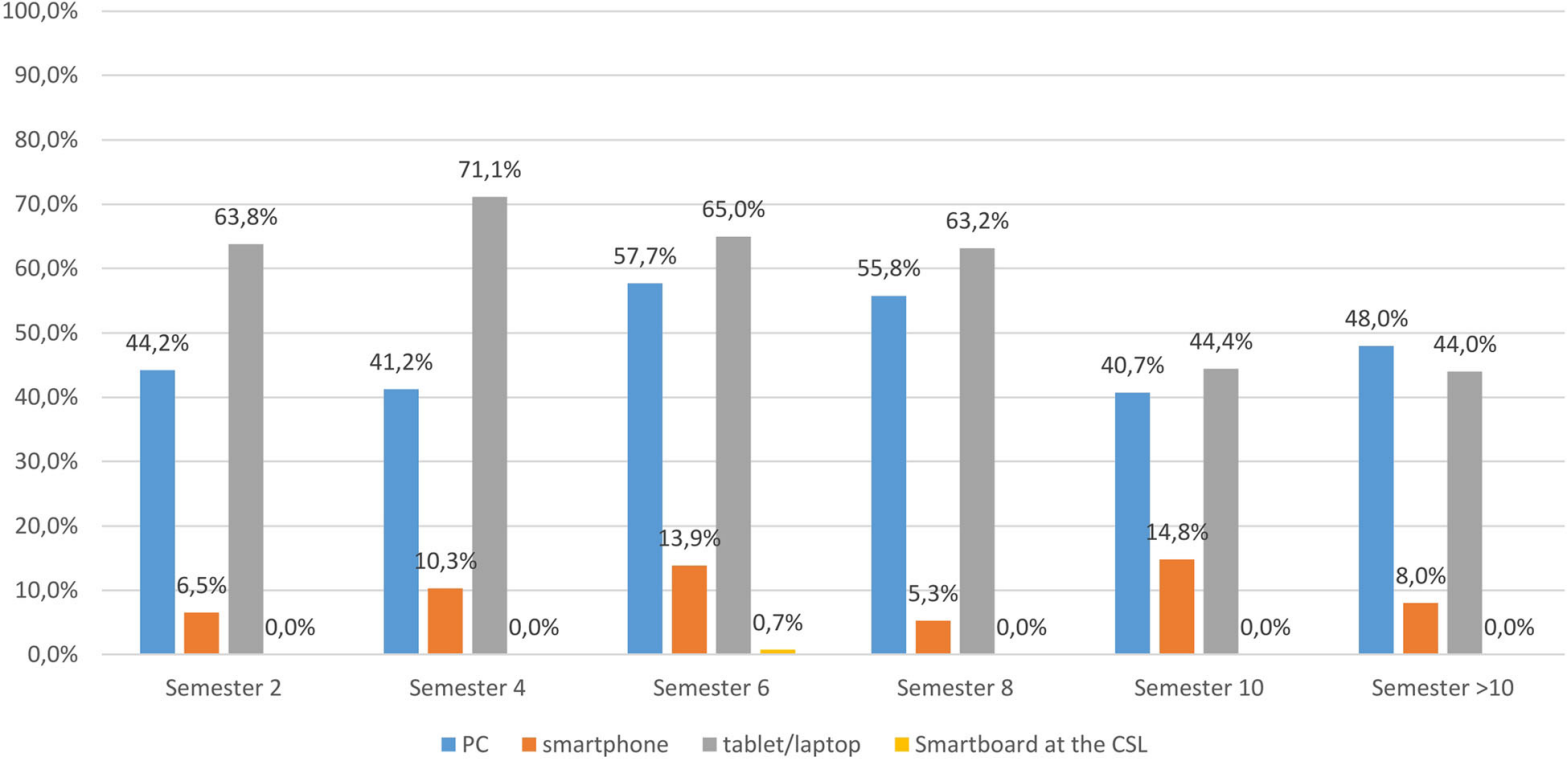
Survey of students at the University of Veterinary Medicine Hannover, Foundation. Question: Which medium do you use to watch videos on TiHoVideos? – multiple answers possible (responses: *n* = 521)

Students mainly used tablets or laptops to view videos on TiHoVideos (63.1%), computers represent the second most frequently used medium (49.5%), (Fig. 5). This question was answered by 62.4% (*n* = 521) of students.

Students had the opportunity to respond openly when asked which additional video topics they would like to see on TiHoVideos. Their responses were compiled into categories: “anatomy” and “situs” (a practical part of the anatomy training using dissection) were most frequently mentioned by second- and fourth-semester students, with second-semester students also identifying “physiology” and “cattle and horses” and fourth-semester students also naming the introductory course for animal handling and restraint, in Germany this course is called “clinical propaedeutics”. The sixth- and eighth-semester students mainly selected “surgery”, “horses”, and “clinical propaedeutics”. In the tenth semester, “surgery” was a popular topic followed by “farm animals”, “surgery/anesthesiology”, and “case reports”. Students in the final semester desired “clinical propaedeutics” and “surgery”.

### Observations

The observations were carried out in the CSL while students were actively using the learning stations. A total of 159 students were observed, of which 145 were female and 14 male. By checking matriculation numbers it was possible to rule out any redundant observations. The following number of students were enrolled in the present study: seventy-two from the second semester, 38 from the fourth, 12 from the sixth, 35 from the eighth and two from the tenth semester. After completing the observed learning stations, the students filled out the second surveys. An instructional video on YouTube was available for 124 of the 159 observed learning stations.

Of the 159 observed students, seven (4.4%) students used a mobile phone during the exercise, it was not possible to discern for all students whether they were watching a video.

#### Survey - observations

All 159 observed students filled out this survey. When asked if they had watched a video on the TiHo YouTube channel (TiHoVideos) covering the selected CSL learning station, 109 students responded in the affirmative (68.6%). Thirty-six students responded in the negative, but would still watch a video (22.6%), 12 said no without wanting to watch such a video (7.5%). Two people did not respond to the question.

**Fig. 6:**
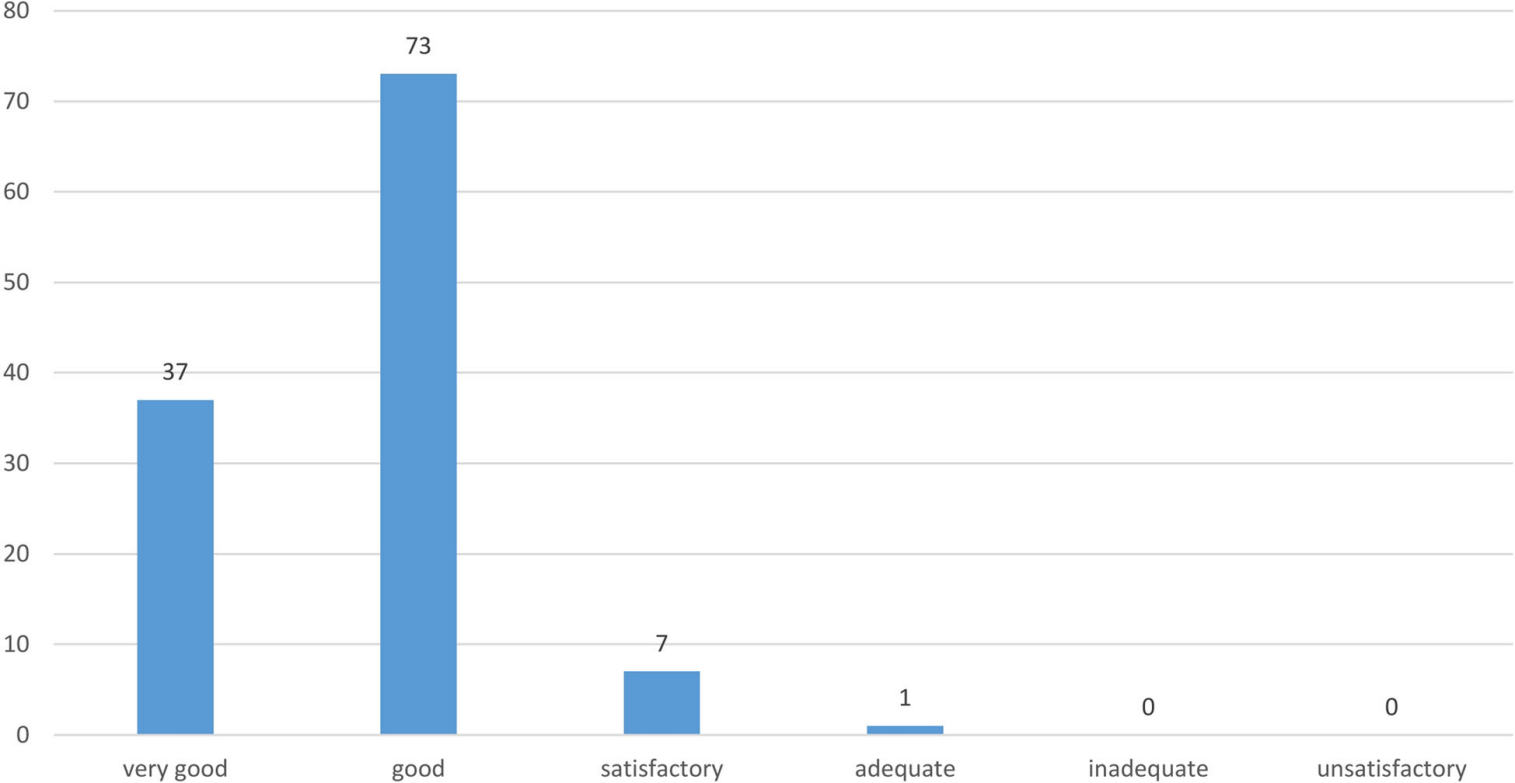
Surveys – Observations of students at the University of Veterinary Medicine Hannover, Foundation (*n* = 159 students). Question: How well did watching the relevant video prepare you for the CSL learning station?

To the question about when they had watched the particular video, 105 (66%) students indicated “at home”, six (3.8%) “mobile”, four (2.5%) “in the CSL” and 45 students (28.3%) said they had not watched any video. Four people did not respond. To the question about where they viewed the videos, 61% (*n* = 97) of the students said “at home on a PC”, 24 (15.1%) “mobile”, two (1.3%) “in the CSL” and two (1.3%) used a “TiHo PC”. Forty-three students did not answer the question. To view videos, 70 (44%) students used a tablet or laptop, 41 (25.8%) a PC, and 20 (12.6%) a smartphone. The Smartboards in the CSL were not used. Forty-one people did not respond to the question. The question about frequency of viewing the particular video was answered by 73 (45.9%) students with “once”, 36 (22.6%) answered with “two to five times”, one person reported watching the videos “more than five times.” A total of 31 (19.5%) responded with “not at all”; 19 people did not answer the question. On average, the students rated the suitability of the videos as preparation for the CSL learning stations with average Ø 1.8 (good) (Fig. 6).

As an additional learning tool, 42 (26.4%) students used literature to prepare for the CSL learning stations. Twenty-five (15.7%) used lecture notes and scripts, four (2.5%) used the instructional videos of other institutions, and 93 (58.5%) students indicated they did not use any additional media.

### Google analytics

User data was analyzed for the period from the YouTube channels’ launch on 26 April 2012 through 31 December 2017. As of June 2018, the TiHoVideos channel currently has 109 videos, 3160 subscribers and approximately 35,000 views per month.

**Fig. 7:**
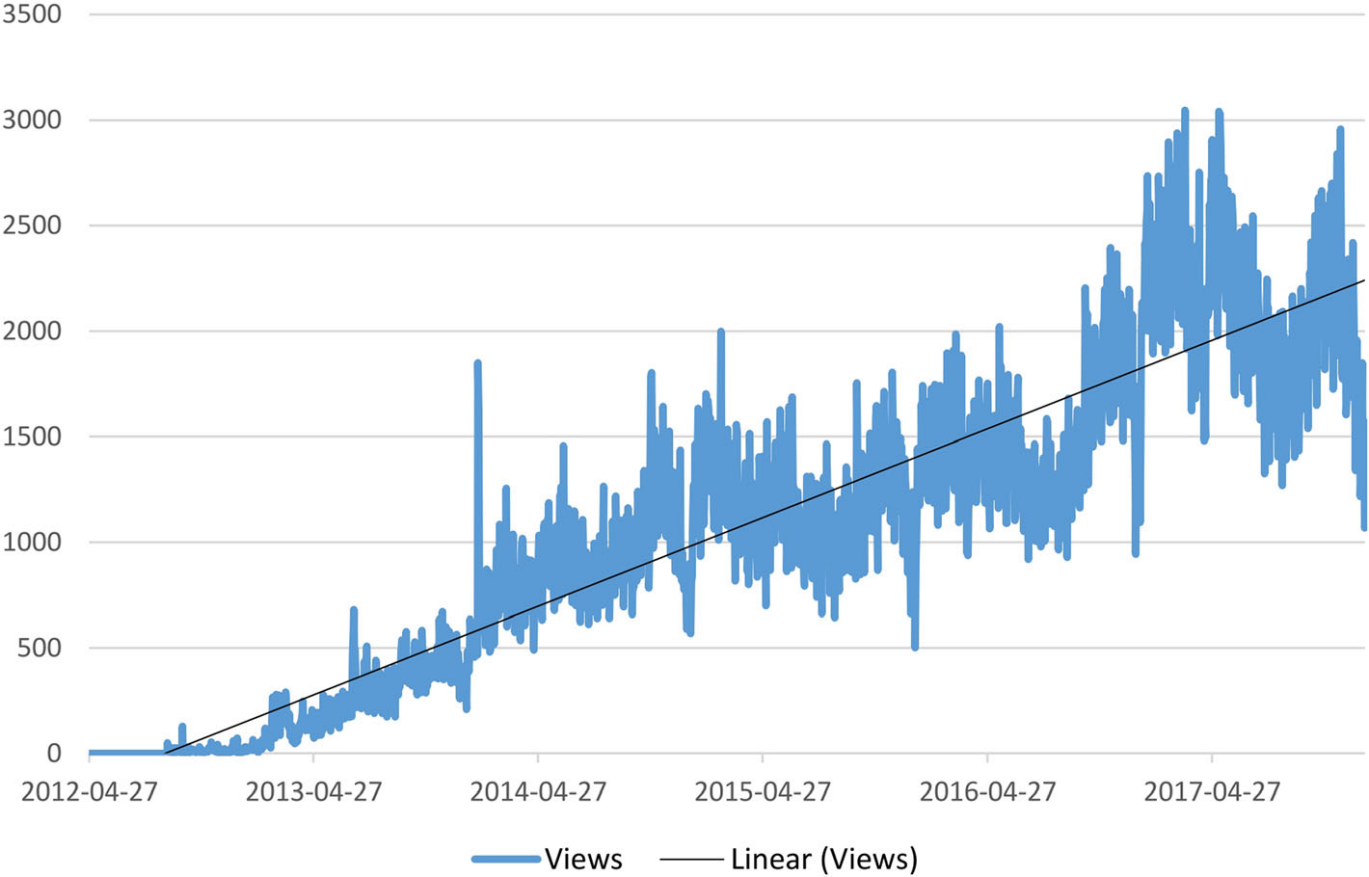
Views of videos on the TiHoVideos channel, time period: 27 April 2012 to 31 Dec. 2017, Google Analytics

The number of views since the channel was launched shows a steady increase (Fig. 7).

**Fig. 8:**
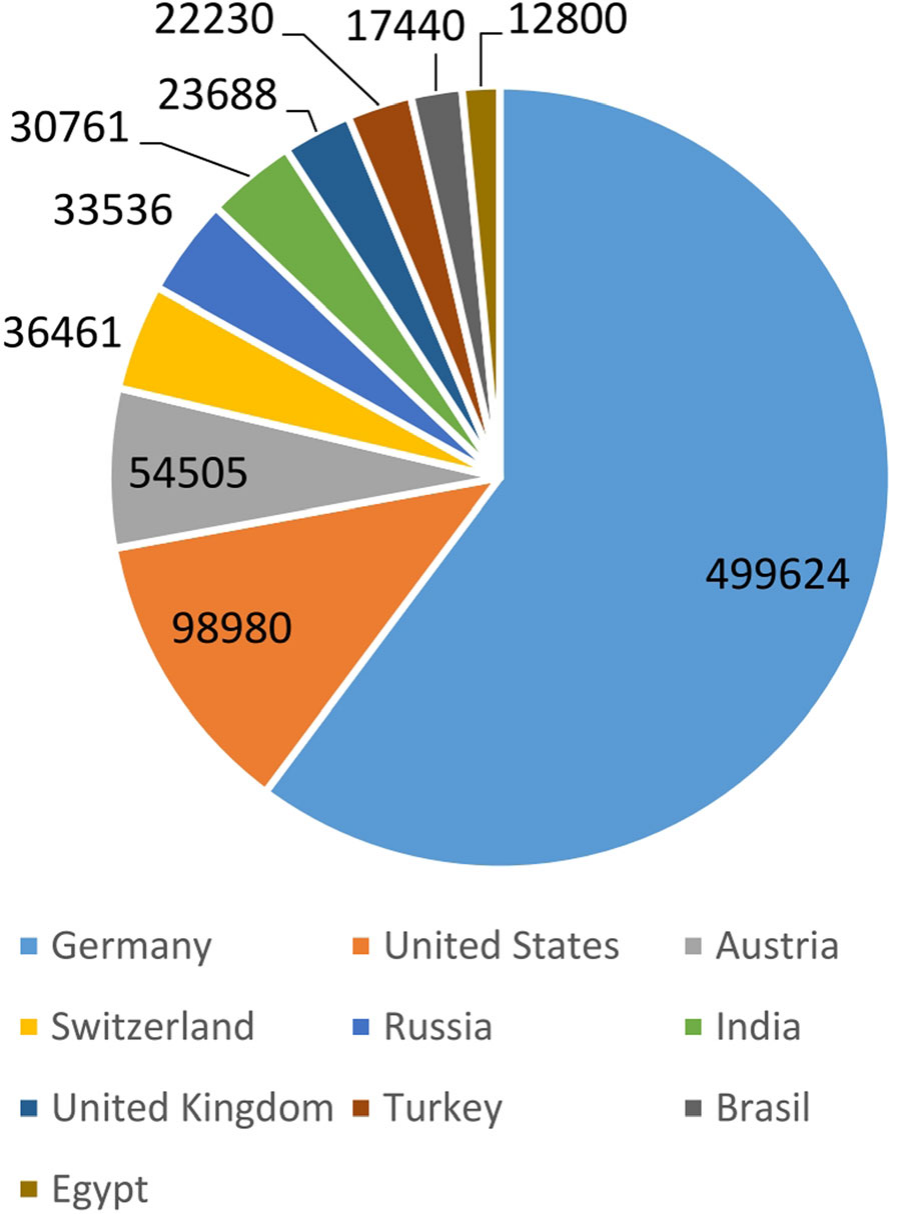
Top ten countries according to views of the TiHoVideos channel, time period: 27 April 2012 to 31 Dec. 2017, Google Analytics

Views are logged for 225 different regions of the world; the top ten are shown in Fig. 8. Most hits come from Germany (45.3%), the United States (9.0%), Austria (4.9%), Switzerland (3.3%) and Russia (3.0%).

The most frequently viewed instructional videos on CSL learning stations are listed in Table 3.

**Table 3:** Top ten videos on the YouTube channel TiHoVideos, Time period: 1 Jan. 2017 to 31 Dec. 2017, Google Analytics

No.	Video title (original title)	Uploaded	Views
1	CSL: Horizontal mattress sutures	24.02.2013	38,457
2	CSL: Vertical mattress suture (Простой линейный обвивной шов)	10.10.2014	32,113
3	CSL: Intracutaneous sutures – continuous stitches (CSL: Intrakutannaht - fortlaufende Naht)	03.03.2014	25,491
4	CSL: Vertical mattress suture pattern	30.04.2013	20,542
5	CSL: Subcutaneous sutures – continuous stitches (CSL: Subkutannaht - fortlaufende Naht)	03.03.2014	19,051
6	CSL: Animal handling and restraint for felines (CSL: Zwangsmaßnahmen und Handling bei der Katze)	13.11.2013	15,593
7	CSL: Open gloving technique	19.12.2013	15,424
8	CSL: Head bandage for dogs and cats - demonstrated on a model	09.10.2013	15,129
9	CSL: Intravenous (I.V.) injection in a dog limb simulator	05.06.2013	14,998

Legend: Time period: 1 Jan. 2017 to 31 Dec. 2017, Google Analytics

Most of the 1,103,732 views for TiHoVideos occurred between 27 April 2012 and 31 December 2017 using computers (49.2%), followed by mobile phones (38.2%), tablets (11.5%), unknown devices (0.5%), televisions (0.4%) and game consoles (0.1%).

### Survey regarding mobile devices

As part of a survey that was sent to TiHo students at the beginning of the project in 2012, questions were also asked about the presence of mobile devices at the time. Of 343 students, 44.6% reported owning a smartphone (*n* = 153), 190 (55.4%) did not possess a Smartphone. A total of 75.2% students with a smartphone used it multiple times a day to access the Internet, 12.4% one per day, 5.9% weekly and 6.5% never. In response to the question regarding which operating systems they used, 52.3% students reported Android, 30.7% iOS, 6.5% Windows, 6.5% other, 3.3% Symbian and 0.7% Black Berry OS.

## Discussion

In order to make the instructional videos accompanying the CSL learning stations easily available to students, the YouTube channel TiHoVideos was created. This YouTube channel currently has approximately 35,000 views per month and over 3100 subscribers with a continuing growth in both numbers (June 2018). The use of the channel was analyzed with Google Analytics. The data that can be collected by Google Analytics are very detailed [4] but cannot lead to any conclusions about the exact origin and background of the users. It also cannot be determined whether or not the users belong to the target group, meaning TiHo students. This is why the students enrolled at the TiHo were surveyed regarding their use of the videos on TiHoVideos.

The use of video material in undergraduate education is promising and should be even further developed [20]. Simulators, models and videos are suitable tools for learning practical skills; the selection of videos should be adapted to the individual learning styles of the students [21]. Video-based e-learning content is clearly superior to text-based materials when imparting practical skills [22] and videos on clinical skills are perceived by students as being useful for learning clinical skills [23]. However, veterinary students have a wide range of individual learning styles, teaching materials should support a broad range of types of learners [24].

Awareness of the TiHoVideos channel exists across all semester levels, on average 63.1% of those surveyed from all semesters knew of the channel, while CSL use is at 46.5% and the use of TiHoVideos at 54.2%. It must be noted that the highest response rate was seen for students in the second semester, a time when there is relatively little time to take advantage of the CSL or the YouTube channel on a regular basis. The results clearly indicate that the majority of students use the CSL and TiHoVideos only after the first preclinical examination (= after the 2nd semester). The average rating of TiHoVideos across all semesters was 2.2 (very helpful) on a six-point Likert scale in reference to its suitability as an additional learning tool.

Across all semesters there is an emphasis at the TiHo on viewing videos on “clinical propaedeutics” (animal handling and restraint) and surgical skills; there is a particular desire in the preclinical second and fourth semesters for topics in anatomy and starting with the sixth semester on the topics of surgery and clinical propaedeutics. The topic of surgery and surgical skills also comes up in the analysis of the user data for TiHoVideos; the five internationally most popular videos demonstrate how to do various suturing techniques properly. The most often cited reasons to watch TiHoVideos were “out of interest” for the second and fourth semesters, and “to prepare for exams” starting in the sixth semester. It is speculated that use in the sixth semester increased due to studying for exams since the content of the YouTube channel still focuses primarily on the clinical phase of study. The use of instructional videos not only fosters theoretical and practical learning, but can also help students perform the exercises with less anxiety [25] and a higher level of confidence [26], a psychological state that is worth striving for in a veterinary program marked by stress [27], particularly during the demanding practical work on living animals [28].

Additionally, according to Gormley [29], videos on clinical skills are rated by students as being the most helpful tool for learning clinical skills. Video instructions for learning stations are the most often offered learning tool for students by different skills labs [30].

According to Google Analytics, access to the videos is primary through computers; no distinction is made in this analysis between PCs and laptops. On the questionnaires, this was approached in a more differentiated manner: the most popular medium used to view videos were tablets or laptops, followed by PCs. Although tablets and laptops are at the top of the list, a clear majority of the students watched TiHoVideos at home.

In a study by Langebaek et al. [31] it was determined that 58% of veterinary students recalled surgical skills via a dynamic visual technique and mentally visualized the relevant instructional videos in order to remember. A third of the students use TiHoVideos to prepare for the CSL.

To investigate the form in which students use instructional videos at the CSL and if the results corroborate the survey responses, student observations were conducted at the CSL after administration of the first questionnaire. The observations enabled discreet determination whether the students viewed instructional videos on site at the relevant CSL learning stations. Most students who were observed in the CSL did not use a mobile phone while working at the learning station; most students indicated that they had prepared for the learning station by watching a video on the TiHoVideos channel. By practicing on models, students are better prepared for their training on living animals [32, 33]. In addition, CSL training supports animal welfare and contributes significantly in veterinary education to optimizing the practical exercises defined as live animal testing [34] under §7a of the German animal protection law [35].

The approach of most skills labs to offer instructional videos as preparation for the learning stations [30] is well-founded and confirms that in Hannover the production of videos for the TiHoVideos channel should be continued in order to make high-quality educational material available to veterinary students on site and around the world.

While less than the half of students at the TiHo possessed a smartphone in 2012, this percentage has, according to a current study by Hildebrandt et al. [36], risen to 94.2% in 2017. Students have fulfilled the technical requirements for using e-learning programs for a number of years [37]. Digitization of teaching is moving forward at the TiHo and can be adapted on an ongoing basis by collecting the appropriate data.

## Conclusions

The provision of instructional videos is in line with contemporary teaching methods. The creation of the YouTube channel, TiHoVideos, by the TiHo Hannover has shown itself to be a good medium for reaching students. By adding more videos on subjects connected with preclinical examinations, the videos will become more attractive to students in the preclinical phase of study. The program could be promoted more heavily since students at all semester levels indicated viewing TiHoVideos for the most part only sporadically. It is to be expected that as a result of this study there has been a clear increase in awareness of TiHoVideos and we can expect a higher user rate from now on.

Although students have the requisite devices, the majority of students observed in the CSL did not use their mobile phones while working at the learning stations. Most students reported having prepared for the learning station by watching a video on the TiHoVideos channel at home. The videos on TiHoVideos are rated as being very helpful learning tools when preparing for CSL learning stations. The instructional videos represent a suitable tool to help veterinary students learn practical skills and are a contribution to animal welfare in undergraduate veterinary education.

## Methods

The utilization of the instructional videos and the user behavior of the students in relation to the instructional videos were measured using questionnaires and augmented by scientific observation of student behaviour during classes at the CSL in combination with a second questionnaire. The questionnaires were developed for this study, see Additional files 1 and 2. In addition, the user data for the YouTube channel and the results of a survey on mobile devices were analyzed. All statistical analysis was performed using descriptive statistics.

### Survey

From July 2015 to January 2016, the questionnaires (Additional file 1) were made available to the 1302 students [38] enrolled at the TiHo, both as paper-based and online surveys (created using SurveyMonkey®, Luxemburg), the online surveys reached students in the part of the curriculum where they would be absent from the University, e.g. to complete practical trainings. The questionnaires first asked students about their use of the CSL and the instructional videos. Then questions were asked about which videos and to what extent these videos were watched and if they represented a helpful learning tool. At the end, students were given an opportunity to make open suggestions for new video topics.

### Observation

In addition, 159 students were observed in the CSL to see how students integrated the videos in their actual practical work. The initially planned sample size of 150 students was based on average CSL user data to that point and was expanded because of convenient samples. Structured, hidden observations by a person that was not participating in the learning station were selected as the method [39], meaning that a standardized observation log was filled in discreetly at set intervals. Usage of mobile devices as well as usage of video material was noted. At the end of a learning station, the observed students were asked to fill out a slightly modified questionnaire (Additional file 2). Observing and surveying the same student twice was ruled out by recording the students’ matriculation numbers.

The data gathered were processed and analyzed anonymously. The data was recorded in Microsoft® Office Excel 2010 (Microsoft Corporation, California, USA) and SurveyMonkey® and analyzed using Microsoft® Office Excel 2010.

### Analysis of utilization

The user data for TiHoVideos were analyzed from its starting date on April 26, 2012 through the end of 2017 using Google Analytics® and Microsoft® Office Excel 2010. This data was relevant to compare global user data to the specific usage by TiHo students.

### Mobile device survey

At the beginning of the project in 2012, an online questionnaire was sent to the students enrolled at TiHo in which information was gathered about which mobile devices the students had in their possession at the time.

## Supplementary information

**Additional file 1:** Survey on the use of YouTube videos. Questionnaire – students enrolled at the TiHo. (DOCX 18 kb)

**Additional file 2:** Survey on the use of YouTube videos (CSL). Questionnaire – students observed at the CSL. (DOCX 14 kb)

## Data Availability

The Datasets used and/or analysed during the current study are available from the corresponding author on reasonable request.

## Abbreviations

CSL: Clinical Skills Lab
TiHo: University of Veterinary Medicine Hannover, Foundation
