# Supplementary material for: TiHoVideos: veterinary students’ utilization of instructional videos on clinical skills
Source: BMC Vet Res. 2019 Sep 11;15:326. doi: 10.1186/s12917-019-2079-2 (PMC6737648; doi:10.1186/s12917-019-2079-2)
Supplement: Supplementary file 1 — Additional file 1: Survey on the use of YouTube videos. Questionnaire – students enrolled at the TiHo. (DOCX 18 kb) [file 12917_2019_2079_MOESM1_ESM.docx]

**Survey on the use of YouTube videos**

1. **Have you already used the CSL?**

□ yes □ no

1. **Are you aware of the TiHo YouTube channel called “TiHoVideos”?**

□ yes □ no

1. **Have you already viewed videos on “TiHoVideos”?**

□ yes □ no

1. **Which videos did you watch?**

*Animal handling and restraint techniques:*

□ Handling, canine I □ Handling, canine II

□ Handling, feline

*Bandaging techniques:*

□ Head bandage □ Paw bandage

□ Bandage thorax/abdomen

*Venipuncture/Injection:*

□ Subcutaneous injection □ Intramuscular injection

□ Intravenous injection in dogs □ Intravenous injection in cats

□ Intravenous injection in model □ Intravenous injection cattle/simulator

□ Insertion of venous catheters and perfusion, cattle

□ Suboccipital puncture to gain cerebrospinal fluid

□ Implantation and reading transponder chips

*First aid:*

□ Intubation in a model □ Resuscitation

*Hygiene/Sterile procedures:*

□ Hand washing □ Hand disinfection

□ Putting on sterile gloves □ Putting on sterile gloves in surgical scrubs

*Surgery/Suturing techniques:*

□ Sterile suture removal □ Removal of needle-thread combination

□ Knot tying techniques □ Instrument knot

□ One-handed surgical knot □ One-handed knot (square knot)

□ Suture technique (Donati) □ Simple interrupted stitch

□ Suture technique (Sultan) □ U-suture

□ Suture technique (Bühner) □ Suture technique (Cushing)

□ Intracutaneous suture □ Suture technique (Lembert)

□ Horizontal mattress suture □ Vertical mattress suture

□ Suture technique (Schmieden) □ Subcutaneous sutures

*Surgical instruments:*

□ Preparing basic surgical instruments □ Holding surgical instruments

□ Scalpel handling □ Surgical instruments, abdominal surgery

*Tutorials:*

□ How to: Start with CASUS □ CASUS registration

*Information on exams:*

□ Electronic assessment at TiHo □ Avoiding cues in multiple-choice questions

*Neurological examinations:*

□ Neurological examination of the dog

□ Neurological examination of the cat

*Mastitis diagnostics:*

□ Udder examination and taking milk samples

□ California Mastitis Test □ Quarter milk samples

*Other:*

□ Study: paraplegic dogs □ The first days of practicing veterinary medicine

□ Feed selection, goats □ Thyroid medicine

□ Seasonal pastures □ Schmallenberg virus

1. **How helpful are the videos on “TiHoVideos” for you when learning?**

□ extremely helpful □ very helpful □ rather helpful

□ rather not helpful □ not helpful □ not at all helpful

1. **How frequently do you watch the videos on „TiHoVideos“?**

□ daily □ weekly □ monthly □ sporadically □ never

1. **Why do you watch videos on “TiHoVideos”?**

□ out of interest □ to prepare for the CSL □ to prepare for exams

□ if it covers a current exam topic

□ other reasons: ____________________________________________________________________________

1. **Where do you watch videos on "TiHoVideos"?**

□ at home on a PC □ mobile □ at the CSL □ TiHo-PC

1. **Which medium do you use to watch videos on “TiHoVideos”?**

□ PC □ Smartphone □ tablet/laptop □ Smartboard at CSL

1. **Which additional video topics would you like to see on “TiHoVideos”?**

____________________________________________________________________________________________________________________________________________________________________________________________________________________________________________________________________________________________________________________________________________________________________________________________

1. **Other comments:**

____________________________________________________________________________________________________________________________________________________________________________________________________________________________________________________________________________________________________________________________________________________________________________________________
