# Supplementary material for: TiHoVideos: veterinary students’ utilization of instructional videos on clinical skills
Source: BMC Vet Res. 2019 Sep 11;15:326. doi: 10.1186/s12917-019-2079-2 (PMC6737648; doi:10.1186/s12917-019-2079-2)
Supplement: Supplementary file 2 — Additional file 2: Survey on the use of YouTube videos (CSL). Questionnaire – students observed at the CSL. (DOCX 14 kb) [file 12917_2019_2079_MOESM2_ESM.docx]

**Survey on the use of YouTube videos (CSL)**

1. **Did you watch a video on the TiHo YouTube channel (TiHoVideos) covering your selected CSL learning station?**

□ yes

□ no, but I will look at it □ no, I will not look at it either

1. **When did you watch the particular video?**

□ at home □ mobile □ in the CSL □ not at all

1. **Where did you watch the particular video?**

□ at home on a PC □ mobile □ in the CSL □ TiHo-PC

1. **Which medium do you use to watch videos on “TiHoVideos”?**

□ PC □ Smartphone □ tablet/laptop □ Smartboard at CSL

1. **How often did you watch the particular video?**

□ 1x □ 2-5x □ >5x □ not at all

1. **How well did watching the relevant video prepare you for the CSL learning station?**

□ very good □ good □ satisfactory □ sufficient □ deficient □ insufficient

1. **Which additional learning tools did you use to prepare for the CSL learning stations?**

□ literature □ lecture notes and scripts

□ instructional videos of other institutions □ none

1. **Which additional video topics would you like to see on “TiHoVideos”?**

____________________________________________________________________________________________________________________________________________________________________________________________________________________________________________________________________________________________________________________________________________________________________________________________

1. **Other comments:**

____________________________________________________________________________________________________________________________________________________________________________________________________________________________________________________________________________________________________________________________________________________________________________________________
